# Targeted Neurotransmitters Profiling Identifies Metabolic Signatures in Rat Brain by LC-MS/MS: Application in Insomnia, Depression and Alzheimer’s Disease

**DOI:** 10.3390/molecules23092375

**Published:** 2018-09-17

**Authors:** Huarong Xu, Zhenru Wang, Lin Zhu, Zhenyu Sui, Wenchuan Bi, Ran Liu, Kaishun Bi, Qing Li

**Affiliations:** 1 School of Pharmacy, National and Local Joint Engineering Laboratory for Key Technology of Chinese Material Medica Quality Control, Shenyang Pharmaceutical University, 103 Wenhua Rd, Shenyang 110016, China; xuh@syphu.edu.cn (H.X.); wangzhenru@163.com (Z.W.); zhulin5122@163.com (L.Z.); iamdanielsui@126.com (Z.S.); liuran8515@outlook.com (R.L.); kaishunbi.syphu@gmail.com (K.B.); 2 China Food and Drug Administration Institute of Executive Development, 16 Xi Zhan Nan Rd, Beijing 100073, China; 3 Health Science Center Department of Pharmacy, Shenzhen University, 3688 Nanhai Ave, Shenzhen 518060, China; cathybcc1106@gmail.com

**Keywords:** insomnia, Alzheimer’s disease, depression, neurotransmitters, LC-MS

## Abstract

Epidemiological, cross-sectional, and prospective studies have suggested that insomnia, Alzheimer’s disease (AD) and depression are mutually interacting conditions and frequently co-occur. The monoamine and amino acid neurotransmitter systems in central nervous system were involved in the examination of neurobiological processes of this symptom complex. However, few studies have reported systematic and contrastive discussion of different neurotransmitters (NTs) changing in these neurological diseases. Thus, it is necessary to establish a reliable analytical method to monitoring NTs and their metabolite levels in rat brain tissues for elucidating the differences in pathophysiology of these neurological diseases. A rapid, sensitive and reliable LC-MS/MS method was established for simultaneous determination of the NTs and their metabolites, including tryptophan (Trp), tyrosine (Tyr), serotonin (5-HT), 5-hydroxyindolacetic acid (5-HIAA), dopamine (DA), acetylcholine (ACh), norepinephrine (NE), glutamic acid (Glu), and γ-aminobutyric acid (GABA) in rat brain tissues. The mobile phase consisting of methanol and 0.01% formic acid in water was performed on an Inertsil EP C18 column, and the developed method was validated well. Results demonstrated that there were significant differences for 5-HT, DA, NE, Trp, Tyr and ACh between model and control group in all three models, and a Bayes linear discriminant function was established to distinguish these three kinds of nervous system diseases by DA, Tyr and ACh for their significant differences among control and three model groups. It could be an excellent strategy to provide perceptions into the similarity and differentia of mechanisms from the point of NTs’ changing in brain directly and a new method to distinguish insomnia, depression and AD from view of essence.

## 1. Introduction

Insomnia, depression and Alzheimer’s disease (AD) are severe diseases of the central nervous system (CNS). Neurotransmitters (NTs) are widely recognized to make the closest contact with CNS diseases. Abnormal alteration of NTs has been found to be nearly concerned to several neurological diseases including insomnia, depression, AD, Huntington’s disease and Parkinsonism etc. [1]. Serotonin (5-HT), a tryptophan-derived biogenic amine, eventually endures oxidative deamination to form 5-hydroxyindolacetic acid (5-HIAA), has been found decreasing significantly in brains of insomnia [2], depression [3] and AD [4] rats. Dopamine (DA) and norepinephrine (NE), the metabolites of tyrosine (Tyr), are involved in sleep-wake cycles, learning, memory and emotion. A number of studies [2,3,4] have shown that the levels of DA and NE decreased significantly in the brains of depression and AD rats, counter to insomnia. Acetylcholine (ACh) is recognized participant in rapid eye movement (REM) sleep [5]. The increasing release of ACh in the pontine reticular formation promoted REM sleep in murine [6,7]. Studies suggested that cholinergic insufficiency contributed to several of the most significant neuropsychiatric appearance of AD [8] and the decreasing breakdown of ACh induced symptoms of anxiety and depression [9]. γ-aminobutyric acid (GABA) and glutamic acid (Glu) are reported as major inhibitory and excitatory NTs, in CNS, respectively. The lack of transformation from Glu to GABA leading strong excitement and weak inhibition is one of causes of some neurological diseases such as insomnia [2], AD [10] and depression [11,12]. It was found that most studies have only investigated if NTs increasing or decreasing in insomnia, depression and AD, respectively, rather than revealing differences and similarities of NTs changes from an overall perspective. So, monitoring neurotransmitter and their metabolite levels in rat brain tissues is an essential tool for examining the similarities and differences in the pathophysiology of these three diseases and also a good method to study the differences in the nature of these interrelated neuropsychiatric disorders.

**Figure 1:**
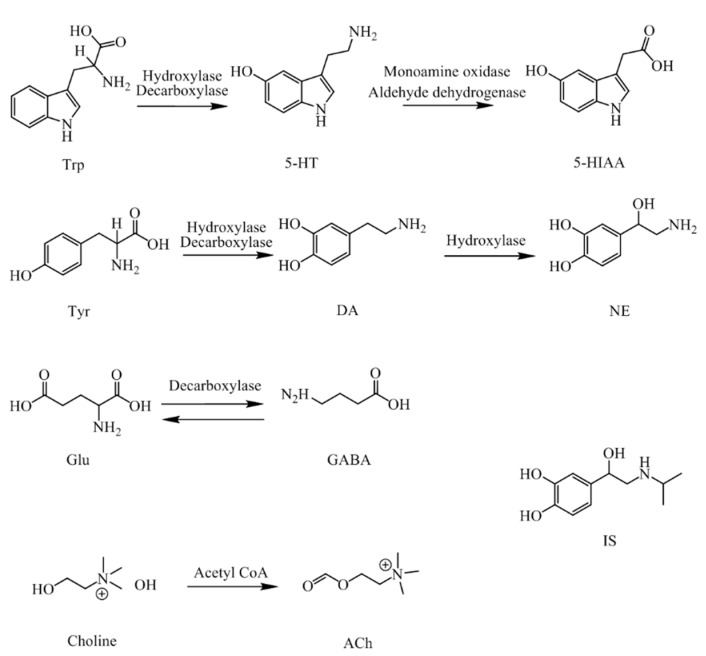
Structures and pathways of the neurotransmitters and their metabolites. (Trp, Tryptophan; 5-HT, Serotonin; 5-HIAA, 5-hydroxyindol acetic acid; Tyr, tyrosine; DA, dopamine; NE, norepinephrine; Glu, glutamic acid; GABA, γ-aminobutyric acid; Ach, acetylcholine; IS, isoprenaline.)

NTs and their metabolites are exist extensively in CNS and the peripheral biofluids of mammals [13,14], including neurotransmitters of amino acid, such as Trp, Tyr, ACh, Glu and GABA, and monoamine neurotransmitters, such as 5-HT, DA, and NE, and also their acidic metabolite 5-HIAA. Their molecular structures and pathways are presented in Figure 1. 

Numerous analytical methods, including high performance liquid chromatography (HPLC) or capillary electrophoresis (CE) involving a series of techniques, such as ultraviolet detection (UV), electrochemical detection (ECD), fluorescence detection (FLD), laser induced fluorescence detection (LIFD) and mass spectrometry (MS), have been engaged in the analysis of NTs in biological samples [15,16,17,18]. Whereas, except the narrow scope of application, all of the analytical procedures have several different drawbacks, such as, low sensitivity and selectivity for UV, poor repeatability and difficulty in simultaneous separation of NTs which have similar electrophoretic behavior, such as Glu and GABA for ECD, and interference after derivatization for FLD. Conventional methods of LC-MS/MS have been used to determine the concentration of NTs with more complex sample preparation, a longer assay time and fewer analytes [19,20]. Therefore, it is necessary to set up a procedure that is simpler, faster and can be used to quantify more analytes simultaneously.

Extracting important features from data is a vital procedure in the issue of pattern recognition, not just can it decrease the computational complexity, but result in better performance through eliminating data redundancy and noise, while overcoming dimension problem for a statistical classifier [21]. As to large category pattern classification, linear supervised feature extraction approach is engaged widely. Bayes linear discriminant method is widely accepted as a good way for the analysis of reliability data, which calculates the between scatter matrix and the within scatter matrix only, then figures out the problem of eigenvalue decomposition easily with efficient computation. To compare different changes of NTs in rat models of insomnia, AD and depression, in our study, a rapid and reliable LC-MS/MS method was established to determinate nine NTs and their metabolites simultaneously in rat brains with a higher sensitivity and shorter chromatographic separation time. The parameters of linearity, precision, recovery and matrix effect were completely validated and successfully applied to analyze the NTs in rat models of insomnia, AD and depression. A Bayes linear discriminant function was established to distinguish these three kinds of nervous system diseases by DA, Tyr and ACh for their significant differences among control and three model groups. The analytical method in this study can also be applied to not only brain issues but also plasma and urine samples, moreover the conclusions of this paper can be used to distinguish insomnia, depression and AD models, which can assist research of neurological diseases. Finally, the marked changes in biomarkers might be applied to clinical diagnosis.

## 2. Results and Discussion

### 2.1. Optimisation of LC-MS/MS Parameters

**Figure 2:**
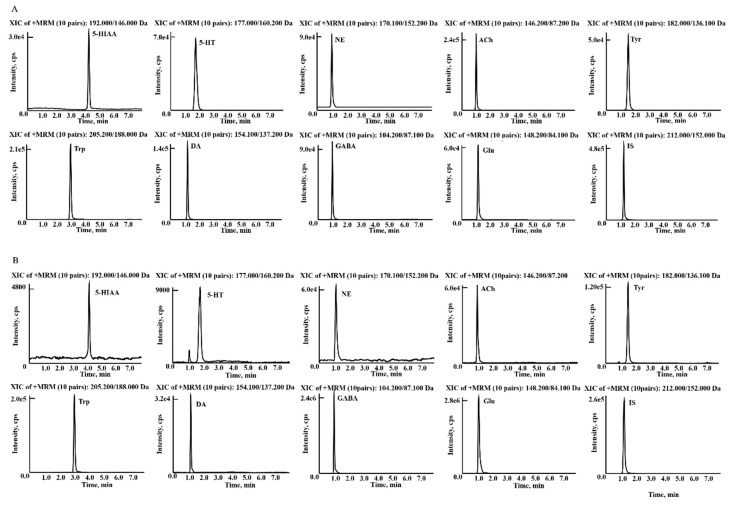
Typical LC-MS MRM chromatograms of (**A**) reference standard solution of the analytes and IS. (**B**) NTs and their metabolites in rat brain samples.

The chromatographic conditions were optimized to deliver higher ionization efficiency and resolution, as well as lower noise of the analytes. In the study, most of the NTs and their metabolites are small molecules with high polarity that show low retainability on C_18_ packed columns and may be co-eluted in the void volume. Therefore, an Inertsil ODS-EP column with optimized gradient elution were engaged to increase their retention and separation. The ODS-EP column was packed with silica gel with polar groups chemically bonded on an octadecyl support, to make sure the analytes cannot be eluted immediately. A signal suppression effect was also avoided, which was suitable for the retention and separation of polar compounds. The methanol-water and acetonitrile-water solvent combination as mobile phase were compared. Methanol was eventually adopted as the organic phase since it showed higher responses and lower noise than acetonitrile. The addition of formic acid could ameliorate the response and peak shapes of the analytes. 0.01% formic acid in water was employed as the aqueous phase after optimization under gradient program with the column temperature setting at 35 °C. Moreover, a tee joint was used to adjust the flow rate of 1.2 mL min^−1^, which could lead to a better solvent vaporization in the MS ionization process. Figure 2 exhibited the representative chromatograms of 5-HT, 5-HIAA, Glu, GABA, NE, DA, ACh, Trp, Tyr and iso-prenaline (IS) in rat brain homogenate samples from a control rat. Ion pairs and different parameters including declustering potentials, entrance potentials, collision energies and cell exit potentials with better response for each analytes were specifically investigated and the MS data were then acquired under multiple reaction monitoring (MRM) mode to obtain the highest quantitative sensitivity by triple quadrupole mass spectrometer.

### 2.2. Sample Preparation

Endogenous metabolites such as NTs are typically small molecules with high polarity. Consequently, a few types of organic solvents were investigated as the deproteinization agent. Acetonitrile and methanol were tested in the first place. It was found that when methanol was used as deproteinization agent, not only did the recoveries give satisfactory results, but also higher response was observed. Secondly, different acids and bases were added into methanol to investigate the influence of different acid-base properties of deproteinization agents including 1% formic acid, 1% ethylic acid and 1% aqueous ammonia. The extraction recovery was closer to 100% within the acceptable range and was more stable when 1% formic acid was added than others. Finally, brain homogenate samples were extracted by methanol with 1% formic acid for protein precipitation.

### 2.3. Method Validation

The NTs studied are all endogenous metabolites which are all already existed in biofluids especially in the rat brain. Therefore, a pooled sample of brain homogenates from the control group (*n* = 8) was prepared using as blank matrix in the method validation process. In the parameters of linearity, recovery and matrix effect, the mean value of each analyte in blank matrix (*n* = 8) was subtracted to eliminate individual differences of rats.

#### 2.3.1. Linearity and LLOQ

The calibration curve was constructed for each analyte by plotting the increased analyte-to-IS peak area ratio (y) between the brain tissue homogenates that spiked standard solutions and the mean ratio of blank samples versus nominal concentration (*x*) by 1/*x*^2^ weighted least square linear regression since the internal standard relative standard derivations (RSDs) of the linearity and precision were less than 15% during the three-day method validation test. Linear regression equations and calibration regression coefficients over concentration ranges of the analytes were shown in Table 1. 

The lower limits of quantification (LLOQ) for each NT in the brain tissue were 1.0 μg mL^−1^ for GABA and Glu, 2.0 ng mL^−1^ for 5-HT and 5-HIAA, 100 ng mL^−1^ for NE and Tyr, and 20 ng mL^−1^ for Trp, DA and ACh respectively, which were also listed in Table 1.

#### 2.3.2. Accuracy and Precision

The accuracy and precision including intra-day precision and inter-day precision of the nine NTs in the rat brain were fully validated and the results were all acceptable (RSD%: <15%; RE%: ±15%). The results were exhibited in Table 2, which indicate an excellent accuracy and precision.

#### 2.3.3. Recovery and Matrix Effect

The mean recoveries of the nine NTs were among 85.0% and 115.0% at different concentration levels, which were also shown in Table 2. The average recovery of the IS was 94.2%, which are within acceptance. The matrix effect of the analytes ranged from 90.0% to 110.0% at three concentration levels (Table 2), while the IS was 96.0%. Results indicated that there were no significant matrix effect interferences for the analytes as well as the IS.

#### 2.3.4. Stability

The concentration measured for the nine analytes at low and high levels deviated by 12.0%, which demonstrated that they were stable in the biosamples at room temperature for 8 h, at 4 °C in the autosampler for 8 h after preparation, 3 freeze-thaw cycles and stored at −80 °C for a month. All the data were within the acceptance and were listed in Table 3.

### 2.4. Method Application

#### 2.4.1. Determination of Neurotransmitters in Rat Brain

The established LC-MS/MS strategy was applied in the target quantification of the NTs in rat brain. Figure 2 shows the chromatograms of the nine analytes and the IS. For more in-depth discussion of a comparative study, the data from the three control groups were combined into one group, since there was no significant difference among the three control groups following a one-way ANOVA using SPSS shown in Table 4. The rat model of *p*-chlorophenylalanine (PCPA)-induced insomnia showed a significant increase of ACh, Tyr, NE and DA, as well as a marked reduction in Glu, 5-HIAA Trp and 5-HT. The levels of 5-HT, 5-HIAA, Glu, DA, NE, ACh, Trp and Tyr were significantly lower in rats with chronic unpredictable mild stress (CUMS)-induced depression. In the AD model rats, the NTs of GABA, 5-HT, DA, NE, ACh, Trp and Tyr showed a significant reduction. In CNS, amino acid and monoamine NTs were transformed into each other. Tyr was converted to Dopa by tryptophan hydroxylase, and then transformed to DA by decarboxylase. DA was converted to NE by DA hydroxylase, followed by transformation to epinephrine (Epi). 

5-OH was also converted from Trp by the effect of hydroxylase and carboxylase, and 5-HIAA was a metabolite of 5-OH and Glu was transformed into GABA by decarboxylase. Regarding the rats in the model of insomnia, the increase in DA may be caused by a significant increase in Tyr, and then more DA is transformed to NE, which is the opposite of what was found in the rat models of depression and AD. ACh, an excitatory neurotransmitter, promote the process of insomnia and reduce depression and AD process. Insomnia, depression and AD may have caused a great increase or decrease in activities of enzyme related to the synthesis and decomposition of ACh, DA and Tyr, which resulted in different changes of these compounds in rat brain. The reduction of 5-HT and 5-HIAA might be attributed to the decrease in Trp or inhibited related enzymes by the treatment methods [22]. The results distinctly indicated that the monoamine and amino acid metabolism pathways were abnormal when the rats were subjected to disposes severally. The remarkable changes of biomarker levels may provide promising understandings in clinical diagnosis of these CNS diseases.

#### 2.4.2. The Comparation of Insomnia, Depression and AD

Except for significant changes in NTs between the control and model groups, DA, Tyr and ACh also exhibited significant differences among the control, insomnia, depression and AD groups. Data from these four groups were further analyzed by Bayes discriminant analysis. If all data were taken for discriminant analysis, only 81.2% of samples could be correctly classified and accuracy of the cross validation was only 77.1%. Low accuracy might be caused by abnormal data; therefore, the data were eliminated reasonably and effectively according to Gobla’s criterion, and accuracy of the discriminant analysis was improved successfully after data optimization. The Bayes linear discriminant function coefficients are shown in Table 5. The 95.3% group samples were correctly classified by this determining equation, and the cross-validation accuracy rate was 86.0%. Using a horizontal comparison, it was found that the absolute value of the coefficient of ACh was larger than the other coefficients in all four discriminant equations, which meant that the change in ACh concentration was most important making a greater impact on the discrimination results. In addition to different changes of NTs, similar rising or decreasing trend were also found. Subjects who have insomnia are 20 times more likely to be depressed than those who do not and, thus, insomnia and depression are interrelated [23]. Similar findings have been reported previously [24,25]. On the other hand, in the study, the correlative symptom of insomnia and depression were presumed to be resulted from commonly lower levels of 5-HT and Glu. Clearly, selective serotonin reuptake inhibitors which increase the concentration of 5-HT in the synaptic cleft are of more befit to patients with depression suffering from insomnia. Since AD is a complicated neurodegenerative disease, the treatment of most AD patients also involves receiving antipsychotic or antidepressant drugs in addition to their actual AD drug to manage the neuropsychiatric and behavioral symptoms [26,27]. The study result demonstrated that the function of DAergic and AChergic nervous systems declined in rats with depression and AD, expressed as reduced concentrations of DA, NE, ACh and Tyr. Monoamine oxidase (MAO) enzyme inhibition, which blocks the oxidation of monoamine NTs such as DA, was a key target for the management of depression and AD, and inhibitors of MAO were the most important drugs for this management. It has been reported that a normal night’s sleep may be critical for maintaining brain health in mice with neurodegenerative diseases, and, most likely, also in humans [28]. Improving sleep quality may help reduce the neurodegenerative risk in old people [29]. Above all, we can propose the following conclusion: DA, Tyr and ACh exhibited significant changes among control and three disease model groups and can be used to distinguish the three kinds of CNS diseases.

## 3. Materials and Methods

### 3.1. Materials and Reagents

5-HT, 5-HIAA, Glu, GABA, DA, NE, ACh, Trp and Tyr were purchased from Sigma (St. Louis, MO, USA). The internal standard isoproterenol (IS) was from the Institute for Food and Drug Control (Liaoning, China). 4-Chloro-D,L-phenylalanine (PCPA), D-galactopyranose (D-gal) and Amyloid β-Peptide Fragment 25–35 (Aβ_25–35_) were also supplied by Sigma-Aldrich (St. Louis, MO, USA). Distilled water was used throughout the experiments. HPLC grade methanol and acetonitrile were from Fisher Scientific (Fair Lawn, NJ, USA). HPLC grade reagents such as acetic acid, formic acid, sodium hydroxide and ammonium hydroxide were provided by Shandong Yuwang Industrial Co., Ltd. (Yucheng, China). Other reagents were all analytical grade.

### 3.2. Animals and Treatment

Forty-eight male wistar rats (200–220 g) were kindly provided by the Experimental Animal Center of Shenyang Pharmaceutical University and bred with unlimited access to food and water in an air-conditioned animal center at a temperature of 22 ± 2 °C and a relative humidity of 50 ± 10%, with a natural light-dark cycle. The animal study was carried out in accordance with the Guideline for Animal Experimentation of Shenyang Pharmaceutical University and the protocol was approved by the Animal Ethics Committee of the institution (Ethic approval document NO. SYPU-IACUC-C2017-1-31-203).

The animals were allowed to acclimatize to the environment for one week before the experiment. Forty-eight rats were randomly divided into six groups, with eight animals each: the insomnia control and model group, the AD control and model group, the depression control and model group. The methods of treating animals used hereinafter are recognized as scientific and effective, widely used [22,30,31,32,33].

Animal model of insomnia was induced by the chemical reagents according to precious report [34]. Briefly, PCPA was suspended in physiological saline. The animal of insomnia model group was administrated intraperitoneal injection of PCPA (350 mg kg^−1^), while the control group was given the same volume of saline solution once a day and last for four days, respectively. After the injection of PCPA, the model group lost their weight and circadian rhythm, and were sleepless all day which proved the success of the model.

The AD model in rats was induced by D-gal and Aβ_25–35_, as described previously [35]. In brief, the rats in AD model and control groups were respectively given intraperitoneal injection of D-gal (50 mg kg^−1^ day^−1^) and the same volume of saline for 6 weeks; then the AD rats were injected with 5 μL Aβ_25–35_ (10 μg μL^−1^) into each bilateral hippocampus at the co-ordinates antero-posterior, −3.5, medio lateral, +2.0, dorso-ventral, 2.8 mm according to the stereotaxic atlas on the fourth week by brain stereotaxic apparatus, individually, while the control rats were administrated the same volume of saline in the same way. After 6 weeks, all rats were trained to find a visible platform in Morris water maze testing. The result demonstrated that the rats in AD model group had a longer time to get to the platform than the control rats at the last trial (*p* < 0.05), which proved the success of the AD model.

The depression model was induced by chronic unpredictable mild stress (CUMS). The model rats were kept separately. Control animals were housed in a separate room and had no contact with the stressed animals. The CUMS procedure was referenced from the precious study [36]. Stressors consisted of (1) 4 h restraint; (2) 24 h wet litter; (3) 24 h food deprivation; (4) 60 s tail pinch; (5) 24 h water deprivation; (6) 5 min cage shake; (7) 12 h cage tilt (cages were tilted to 45° from the horizontal). In this respect, stressors were administrated in a semi-random manner at any time of day, and the stress sequence was changed every week in order to make the stress procedure unpredictable. After 3 weeks the autonomous behavior of rats was evaluated by the Open-field test (OFT). The result demonstrated that the vertical and horizontal bouts of rats in depression model group were lower than the control group (*p* < 0.05), which proved the success of the CUMS model.

### 3.3. Instruments and LC–MS/MS Conditions

The LC-ESI-MS/MS system was performed using an LC-20 A Prominence™ UFLC XR system consisted of a binary pump, a degasser, an autosampler and a thermostatted column compartment (Shimadzu, Kyoto, Japan); a 4000 QTRAP™ triple quadrupole-linear ion trap mass spectrometry system equipped with a turbo ion spray source (Sciex, Foster City, CA, USA). All the operations, the acquiring and analysis of data were controlled by Analyst software (version 1.6, Sciex).

Separations were accomplished on an Inertsil ODS-EP column (4.6 mm × 150 mm, 5.0 μm) (GL Sciences, Tokyo, Japan) protected by a high-pressure column pre-filter (2 mm) at 35 °C. The mobile phase consisting of 0.01% formic acid in water (A) and methanol (B) was delivered at a flow rate of 1.2 mL min^−1^ with one third of the eluent splitted into the inlet of mass spectrometer. The linear gradient elution program was as follows: 0–0.5 min, 15% B; 0.5–5 min, 15–85% B; 5–6 min, 85% B; 6–8 min, 15% B. The injection volume was 4 μL and the total time taken for the chromatographic run was 8.0 min. The analytes and IS were ionized by the ESI source in positive ion mode and the ion spray voltage was set at 5500 V. The curtain gas, gas 1 and gas 2 (nitrogen) were set at 20 psi, 50 psi and 50 psi with a source temperature of 500 °C. Quantitative MRM ion pairs parameters are listed in Table 6.

### 3.4. Standard Solution and Quality Control Samples

Standard stock solutions of 5-HT, 5-HIAA, Glu, GABA, DA, NE, ACh, Trp, Tyr and IS were separately prepared in methanol-water (20:80, *v/v*). The stock solutions of the analytes were further diluted with methanol-water (20:80, *v/v*) to make a series of mixed working standard solutions at the desired concentrations. The brain tissue samples were homogenized in a twenty-fold volume of methanol with 1% formic acid. The brain tissue standards of analytes for each of the nine analytes were prepared as follows, GABA and Glu at concentrations of 1, 2, 4, 8, 16 and 32 μg mL^−1^, 5-HT and 5-HIAA at concentrations of 2, 4, 8, 16, 32 and 64 ng mL^−1^, NE and Tyr at concentrations of 100, 200, 400, 800, 1600 and 3200 ng mL^−1^, Trp, DA and ACh at concentrations of 20, 40, 80, 160, 320 and 640 ng mL^−1^. These standards were prepared by adding appropriate amount of standard working solutions to blank brain tissue homogenates. Quality control samples were prepared using the same method (4, 16 and 50 ng mL^−1^ for 5-HT and 5-HIAA; 2, 8 and 25μg mL^−1^ for Glu and GABA; 200, 800 and 2500 ng mL^−1^ for NE and Tyr; 40, 160 and 500 ng mL^−1^ for Trp, DA and ACh. A working solution for the IS (10 μg mL^−1^) was also prepared.

### 3.5. Sample Preparation

All rats were sacrificed by decapitation without anesthesia. The brains were rapidly removed above ice bath, frozen and stored at −80 °C until extraction. All frozen samples, calibration standards and QC samples were thawed and allowed to equilibrate at room temperature prior to analysis. The frozen brain tissue samples were dissected and homogenized in a twenty-fold volume of methanol with 1% formic acid above ice-water bath. The homogenates were centrifuged at 12,000 × *g* for 20 min at 4 °C. Then, 100 μL of the supernatant was transferred to a 1.5 mL centrifuge tube, spiked with 10 μL of the IS solution and 10 μL methanol, followed by vortexing for 30 s. Then 100 μL methanol was added into the supernatant, followed by vortexing for 1 min. Next, these samples were centrifuged at 12,000× *g* for 5 min at 4 °C. Finally, a 4 μL aliquot was injected into the LC-MS/MS system for analysis.

### 3.6. Method Validation

The analytical method validation procedure was in accordance with the US Food and Drug Administration Bioanalytical Method Validation Guidance for Industry [37]. Validation parameters included lower limit of quantification (LLOQ), linearity, precision, accuracy, recovery, matrix effect and stability.

Neurotransmitters are endogenous substances which already existed inherently in biosamples, especially brain homogenates. Therefore, blank value of each analyte should be subtracted from each calibration point. The calibration curve was constructed for each analyte by plotting the increased analyte-to-IS peak area ratio (*y*) between the brain tissue homogenates that spiked standard solutions and the mean ratio of blank samples versus nominal concentration (*x*) by *1/x*^2^ weighted least square linear regression. LLOQ was defined as the lowest concentration of the calibration curve with acceptable accuracy and precision, which provided an intensity of signal-to-noise ratio above 10.

The validation of accuracy and precision and were performed with six replicates at three (low, medium and high) QC concentration levels on the same day and on three consecutive days. Precision was presented as the relative standard deviation (RSD %), while accuracy was presented as relative error (RE%).

The extraction recovery for each analyte was calculated by comparing the difference of the responses between the spiked sample before and after extraction at three QC levels, while the mean value of blank samples was removed.

The matrix effect was evaluated as follows: the analyte-to-IS peak area ratio for each analyte in the spiked sample after extraction subtracted the mean ratio of blank samples at three concentration levels of QC samples, and then the increased peak area ratio was divided by the mean ratio measured in corresponding standard solutions.

Stability tests were conducted on triplicate at low and high QC levels under the storage conditions as follows: 8 h at room temperature, 4 °C in the autosampler for 8 h after preparation, three freeze-thaw cycles and stored at −80 °C for three months.

### 3.7. Statistical Data Analysis

All data are illustrated as the mean ± SD. The acquired data were analyzed by ANOVA and Bayes discriminant analysis using the SPSS 20 statistical software (IBM, Armonk, NY, USA). A *p* value less than 0.05 was considered statistically significant for all the tests.

## 4. Conclusions

In this paper, a simple, fast and sensitive LC-MS/MS method using an ODS-EP column, without derivatization or ion-pairing reagents for simultaneous determination of nine NTs in rat brain was established and fully validated. Following optimization of the chromatographic conditions, the compounds were found to have symmetric peak shapes and could be assayed in 8.0 min, which was very suitable for high throughput bioanalysis. A simple sample preparation involving extraction was applied and the recoveries of all the analytes above 85.0%. In addition, we discovered changes in several NTs and their metabolites in rats with insomnia, depression and AD, and established a Bayes linear discriminant function to differentiate these three kinds of neurological diseases. Results indicated that there were significant differences for 5-HT, DA, NE, Trp, Tyr and ACh between model and control group in all three models, what is more, DA, Tyr and ACh showed significant differences among control and three model groups and were used to distinguish these three kinds of nervous system diseases successfully with a cross-validation accuracy rate 86.0%.The data obtained in this study make a promising understanding to the diagnosis and treatment of insomnia, depression and AD.

## Figures and Tables

**Table 1:** Linear ranges, regression equations, correlation coefficients and Lower Limit of Quantitation (LLOQ) of multicomponent in rat brain homogenates.

Analyte	Linear Regression Equation	Linear Range (ng/mL)	Correlation Coefficient (R^2^)	LLOQ	
				Accuracy (RE%)	Precision (RSD%)
5-HT	y = 3.675 × 10^−4^ x + 9.17 × 10^−4^	2.0~64	0.9979	10.8	11.5
5-HIAA	y = 1.568 × 10^−4^ x + 9.85 × 10^−4^	2.0~64	0.9920	−5.9	10.2
Glu	y = 1.617 × 10^−1^ x + 1.954	1.0 × 10^3^~3.2 × 10^4^	0.9928	3.4	8.1
GABA	y = 2.792 × 10^−1^ x + 1.325	1.0 × 10^3^~3.2 × 10^4^	0.9935	−6.9	7.8
DA	y = 8.126 × 10^−4^ x + 1.353 × 10^−3^	20~6.4 × 10^2^	0.9983	11.9	9.7
ACh	y = 1.802 × 10^−3^ x + 1.571 × 10^−2^	20~6.4 × 10^2^	0.9976	7.9	10.5
Trp	y = 1.063 × 10^−3^ x + 1.042 × 10^−1^	20~6.4 × 10^2^	0.9961	7.9	3.7
Tyr	y = 6.268 × 10^−4^ x + 4.080 × 10^−2^	1.0 × 10^2^~3.2 × 10^3^	0.9944	9.1	6.3
NE	y = 1.536 × 10^−3^x + 2.270 × 10^−2^	1.0 × 10^2^~3.2 × 10^3^	0.9974	−7.4	5.6

RE, relative error; RSD, relative standard derivations.

**Table 2:** Summary of accuracy, precision, recovery and matrix effect of the nine analytes in rat brain (*n* = 8).

Analytes	Concentration (ng mL^−1^)	Intra-Day RSD (%)	Inter-Day RSD (%)	Accuracy (RE%)	Recovery (%, mean ± SD)	Matrix Effect (%, mean ± SD)
5-HT	4	3.1	3.6	−11.4	105.6 ± 7.4	98.5 ± 9.5
	16	5.2	1.6	−9.0	99.0 ± 9.4	98.0 ± 1.9
	50	4.8	7.2	7.3	102.0 ± 8.8	98.7 ± 2.0
5-HIAA	4	8.9	14.7	1.5	97.0 ± 6.7	92.2 ± 8.6
	16	7.8	13.7	4.2	95.5 ± 6.5	92.2 ± 6.3
	50	6.7	10.6	7.0	97.5 ± 7.2	90.2 ± 5.7
Glu	2000	6.5	2.5	3.0	102.9 ± 9.8	101.4 ± 8.5
	8000	4.4	1.0	8.4	99.5 ± 6.4	95.8 ± 4.4
	25,000	3.9	4.4	10.9	98.9 ± 9.9	97.1 ± 5.9
GABA	2000	6.9	6.0	−1.7	102.2 ± 8.1	95.4 ± 10.8
	8000	4.9	5.8	2.3	105.1 ± 4.7	98.3 ± 5.2
	25,000	9.4	7.4	12.9	92.6 ± 9.0	99.2 ± 4.0
DA	40	5.4	3.2	8.0	98.1 ± 8.0	90.1 ± 6.8
	160	2.9	2.1	12.0	95.2 ± 7.9	94.6 ± 3.8
	500	13.4	0.7	13.4	94.4 ± 6.8	99.8 ± 5.4
NE	200	6.4	2.8	12.9	96.4 ± 9.5	102.2 ± 7.0
	800	5.4	4.9	−13.4	90.6 ± 4.3	104.3 ± 6.2
	2500	7.9	5.4	4.3	95.0 ± 5.2	97.0 ± 4.7
ACh	40	5.4	1.2	4.6	92.3 ± 12.5	99.5 ± 7.7
	160	2.4	3.1	12.6	95.5 ± 9.4	91.3 ± 3.6
	500	11.4	7.0	9.3	96.9 ± 7.8	103.4 ± 2.3
Trp	40	9.4	3.5	−5.4	93.2 ± 9.8	101.0 ± 9.7
	160	7.2	2.2	1.7	101.4 ± 8.0	96.9 ± 6.2
	500	4.1	0.8	4.1	98.9 ± 6.0	101.9 ± 5.6
Tyr	200	6.0	7.1	−1.9	95.5 ± 8.9	102.4 ± 6.0
	800	3.2	6.2	7.8	98.6 ± 7.8	101.4 ± 1.7
	2500	4.2	4.2	8.8	96.7 ± 6.5	97.3 ± 7.9

5-HT, Serotonin; 5-HIAA, 5-hydroxyindol acetic acid; Glu, glutamic acid; GABA, γ-aminobutyric acid; DA, dopamine; NE, norepinephrine; Ach, acetylcholine; Trp, Tryptophan; Tyr, tyrosine; RE, relative error; RSD, relative standard derivations.

**Table 3:** Stability of analytes at different conditions determined by LC-MS/MS (RE%, *n* = 3).

Analytes	Concentration (ng mL^−1^)	8 h, 4 °C		8 h, Room Temperature		3 Freeze-Thaw Cycles		−80 °C for 3 Months	
		RE (%)	RSD (%)	RE (%)	RSD (%)	RE (%)	RSD (%)	RE (%)	RSD (%)
5-HT	4	3.0	4.5	−4.9	3.3	−2.9	4.1	4.2	2.7
	50	1.3	2.0	−2.1	2.3	0.8	3.0	−1.0	5.7
5-HIAA	4	−9.3	1.6	−9.0	4.5	2.3	1.0	1.0	2.2
	50	−7.4	7.4	−3.7	5.2	−1.1	6.9	−4.6	2.3
Glu	2000	−5.0	3.6	−5.9	6.0	−2.0	1.8	4.1	6.4
	25,000	8.5	5.2	6.6	3.7	−2.0	6.3	−6.6	3.5
GABA	2000	−4.0	2.6	3.8	2.2	3.1	1.4	1.5	1.9
	25,000	2.7	4.6	6.7	1.6	5.9	6.6	−3.1	1.5
DA	40	5.7	9.5	-9.6	8.8	1.7	0.7	−2.8	5.8
	500	2.3	7.1	1.9	6.6	−1.2	5.0	4.5	4.2
NE	200	−3.2	7.2	2.7	5.0	−2.7	2.8	−2.5	3.6
	2500	−3.2	5.0	−4.7	2.1	3.6	2.8	−1.2	2.9
ACh	40	8.7	4.4	−5.8	2.4	−2.9	1.7	2.2	1.2
	500	−6.0	5.6	−1.5	2.5	−2.6	7.7	−5.3	1.6
Trp	40	1.1	3.2	11.4	5.5	2.7	2.1	−1.9	0.9
	500	−3.9	7.0	5.9	2.7	1.1	4.7	−0.9	2.4
Tyr	200	2.6	3.8	2.9	5.8	5.1	3.7	−2.7	2.1
	2500	−7.8	3.5	−5.7	6.0	2.1	5.4	−1.2	1.3

5-HT, Serotonin; 5-HIAA, 5-hydroxyindol acetic acid; Glu, glutamic acid; GABA, γ-aminobutyric acid; DA, dopamine; NE, norepinephrine; Ach, acetylcholine; Trp, Tryptophan; Tyr, tyrosine; RE, relative error; RSD, relative standard derivations.

**Table 4:** The concentrations of NTs and their metabolites in rat brain samples.

Analyte (ng mg^−1^)	Control Group	Insomnia Group	Depression Group	AD Group
5-HT	1.551 ± 0.262	0.449 ± 0.113 ^aa^	0.940 ± 0.061 ^aa, bb^	0886 ± 0.087 ^aa,bb^
5-HIAA	0.960 ± 0.263	0.556 ± 0.135 ^aa^	0.324 ± 0.121 ^aa^	0.766 ± 0.306 ^c^
Glu	1219.476 ± 94.828	948.926 ± 144.407 ^aa^	988.866 ± 161.352 ^a^	1288.012 ± 56.815 ^bb,cc^
GABA	861.936 ± 83.004	840.986 ± 139.038	896.462 ± 71.449	623.404 ± 140.555 ^a,bb,cc^
DA	7.225 ± 0.873	10.315 ± 0.824 ^aa^	4.022 ± 0.972 ^aa,bb^	5.353 ± 1.016 ^aa,bb,cc^
NE	22.616 ± 3.435	53.086 ± 3.267 ^aa^	19.771 ± 2.776 ^a,bb^	17.602 ± 2.372 ^aa,bb^
ACh	3.787 ± 0.506	5.059 ± 0.590 ^aa^	2.510 ± 0.273 ^aa,bb^	3.132 ± 0.322 ^aa,bb,c^
Trp	21.837 ± 2.311	19.405 ± 2.265 ^a^	18.135 ± 1.769 ^aa^	14.791 ± 1.721 ^aa,bb,cc^
Tyr	39.254 ± 5.862	74.728 ± 10.452 ^aa^	19.644 ± 2.120 ^aa,bb^	29.533 ± 6.896 ^a,bb,c^

For statistical significance ^a^ *p* < 0.05, ^aa^ *p* < 0.01 compared with control group, ^bb^ *p* < 0.01 compared with insomnia group, ^c^ *p* < 0.05, ^cc^ *p* < 0.01 compared with depression group.

**Table 5:** Bayes linear discriminant function coefficients of DA, Tyr, ACh and constant.

	Group			
	Blank	Insomnia	Depression	AD
DA	7.740	10.723	4.310	5.692
ACh	16.614	21.920	11.152	13.836
Tyr	0.760	1.533	0.367	0.571
Constant	−75.047	−169.858	−27.946	−47.148

**Table 6:** List of selected MRM parameters, declustering potential (DP), entrance potential (EP), collision energy (CE) and cell exit potential (CXP) for each analyte and IS (Isoprenaline).

Analyte	Q1 Mass (Da)	Q3 Mass (Da)	DP	EP	CE	CXP
5-HT	177.1	160.2	38	10	10	7
5-HIAA	192.0	146.0	25	9	20	8
Glu	148.2	84.1	41	10	24	15
GABA	104.2	87.1	26	14	15	16
DA	154.1	137.2	37	100	13	8
NE	170.1	152.2	33	5	11	9
ACh	146.2	87.2	52	3	20	4
Trp	205.2	188.0	40	4	14	12
Tyr	182.0	136.1	52	8	25	7
IS	212.0	152.0	46	8	20	9
